# Draft genome sequence of *Aspergillus flavus* isolate TERIBR1, a highly tolerant fungus to chromium stress

**DOI:** 10.1186/s13104-019-4484-9

**Published:** 2019-07-19

**Authors:** Pushplata Prasad Singh, Akanksha Jaiswar, Divya Srivastava, Alok Adholeya

**Affiliations:** TERI-Deakin Nanobiotechnology Centre, TERI Gram, The Energy and Resources Institute, Gwal Pahari, Gurgaon Faridabad Road, Gurgaon, Haryana 122 001 India

**Keywords:** *A. flavus* isolate TERIBR1, Whole genome sequencing, de novo assembly

## Abstract

**Objectives:**

*Aspergillus flavus* isolate TERIBR1 was isolated from tannery sludge highly contaminated with chromium. During characterization process, it exhibited capability to adapt and grow in fungal growth media amended with chromium concentration as high as 250 mg/l. In order to understand the genetic underpinnings of the chromium tolerance trait, whole genome sequencing of the TERIBR1 genome was carried out. Information from the current genome will facilitate an understanding of the mechanisms underlying fungal adaptation to heavy metal stress and also heavy metal bioremediation.

**Data description:**

Here, we report the draft genome sequence along with the assembly and annotation methods used for genome sequence of the *A. flavus* isolate TERIBR1. The draft genome assembly size is estimated at 37.7 Mb coding for 13,587 genes and has high similarity to the reference genome of *A. flavus* strain NRRL3357.

## Objective

Several species of filamentous fungi have been identified for their bioaccumulation or biosorption potentials [1–4]. Reduced cost and environmental-toxicity through microbial bioremediation approach makes it favorable over the conventional methods [5]. The genome of several *A. flavus* strain have been reported previously https://www.ncbi.nlm.nih.gov/genome/?term=aspergillus+flavus). The ability of the *A. flavus* isolate TERIBR1 to adapt and grow in tannery sludge highly contaminated with chromium inspired us to carry out its whole genome sequencing. The genome sequence reported here was utilized for comparative genomics study to understand the putative influence of the abundantly present non-synonymous SNP in TERIBR1 on the function of candidate genes involved in chromium tolerance [6].

## Data description

Pure culture of *A. flavus* isolate TERIBR1 was recovered through an enrichment culture technique from tannery sludge [containing very high concentration of of Cr(III)] and molecularly characterized by the universal fungal primer set for Ascomycetes (ITS1: 5′ TCCGTAGGTGAACCTGCGG, 3′ (Eurofins India, Cat. No. 24-1023-5/6) and ITS4A: 5′ CGCCGTTACTGGGGCAATCCCTG 3′ (Eurofins India, Cat. No. 24-2002-1/6). Genomic DNA was extracted using the DNeasy plant maxi kit (QIAGEN, USA; cat. No. 68163). Using a whole-genome shotgun approach, two TruSeq paired-end (PE) libraries (insert sizes 180 bp and 500 bp) and a mate pair (MP) library (insert size ~ 5 Kb) was generated. An Illumina (HiSeq 2000) machine at a commercial facility (MOgene LC, USA) was used for sequencing. DNA libraries were loaded into Illumina flow-cells at concentrations of 1.4–1.75 pM. Cluster generation was performed in a cBOT automated cluster generation system. Real Time Analysis (RTA) software (rta_1–13) was used to process the image analysis and base calling. Sequencing of the DNA libraries yielded 5.4 Gb of PE reads and 2.6 Gb of MP reads. The raw reads were trimmed using Trimmomatic V 0.36 [7]. Quality-passed reads were assembled using the de novo genome assembler ALLPATHS-LG. PE reads with overlaps were first combined to form contigs. MP reads were used for gap filling in order to get sequences with minimal N’s and the longest length. Table 1 presents webpage links for genome assembly and annotation data files. The resulting 3,77,32,467 bp (100 X coverage) draft genome assembly [10] comprises of 322 contigs greater than 900 bp and has an *N*50 of 1,536,000 bp and an *L*50 of 9 contigs (Additional file 1). The GC content of the assembled genome is 48.30%. 225 out of 248 ultra-conserved eukaryotic genes were identified in the assembly through CEGMA ([8], Additional file 2). The MAKER v2.31.9 [9] genome annotation and curation pipeline predicted 13,587 protein coding genes as compared to 13,659 in NRRL3357. Using blastp search in the NCBI NR database, significant matches were identified for 11,120 protein-coding genes. An InterProScan analysis was also performed in order to further annotate the predicted genes with protein functional domains. 2551 proteins with InterProScan domains were identified (Additional file 3); major protein families included, Major facilitator superfamily (n = 334), fungal specific transcription factor domain (n = 190), Cytochrome P450 (n = 140), sugar (and other) transporters (n = 127), Protein kinase domain (n = 112), short chain dehydrogenase (n = 112) and fungal Zn(2)-Cys(6) binuclear cluster domain (n = 94) (Additional file 4). Genes were also annotated by using Blast2GO V5 basic [10] based on the term “biological function” in Gene Ontology (GO) (Additional file 5).

**Table 1:** Overview of data files

Label	Name of data file	File types	Data repository
BioProject [10]	Genome Assembly of TERIBR1	.fasta file	https://www.ncbi.nlm.nih.gov/bioproject/PRJNA362980/
Additional file 1 [11]	*Aspergillus flavus* TERIBR1 assembly scaffolds length	.xls	10.6084/m9.figshare.8325338
Additional file 2 [12]	*Aspergillus flavus* genome completeness statistics based on 248 CEGs	.xls	10.6084/m9.figshare.8325335
Additional file 3 [13]	Interproscan domains in *Aspergillus flavus* TERIBR1	.xls	10.6084/m9.figshare.8325347
Additional file 4 [14]	GO based genes biological functional annotation domains present in *Aspergillus flavus* (TERIBR1).	.tif	10.6084/m9.figshare.8325344
Additional file 5 [15]	Top 20 InterPro domains distribution in the genome repertoire of TERIBR1 and NRRL3357	.tif	10.6084/m9.figshare.8325341

## Limitations

Illumina sequencing reads generated in this study were de novo assembled and annotated to understand the gene/protein repertoires in the chromium tolerant isolate of *A. flavus*. Since the whole genome sequencing project involved use of both PE and MP libraries for scaffold development, a high quality assembly with 100 X coverage could be generated. Therefore, we did not notice any serious limitations of the data.

## Data Availability

Genomic assembly of *A. flavus* TERIBR1; https://www.ncbi.nlm.nih.gov/bioproject/PRJNA362980/. The additional files (Additional File 1, Additional File 2, Additional File 3, Additional File 4, Additional File 5) can be accessed openly on Figshare (https://figshare.com)

## Abbreviations

nsSNPs: non-synonymous single nucleotide polymorphism
PE: paired-end
MP: mate pair
